# Experimental and Numerical Simulation on the Penetration for Basic Magnesium Sulfate Cement Concrete

**DOI:** 10.3390/ma16114024

**Published:** 2023-05-28

**Authors:** Qiquan Mei, Hongfa Yu, Haiyan Ma, Yongshan Tan, Zhangyu Wu

**Affiliations:** 1 Department of Civil and Airport Engineering, Nanjing University of Aeronautics and Astronautics, Nanjing 210016, China; meiqiquan@nuaa.edu.cn (Q.M.); mahaiyan@nuaa.edu.cn (H.M.); 2 College of Civil Science and Engineering, Yangzhou University, Yangzhou 225127, China; ystan@nuaa.edu.cn; 3 Jiangsu Key Laboratory of Construction Materials, School of Materials Science and Engineering, Southeast University, Nanjing 211189, China; wuzy@nuaa.edu.cn

**Keywords:** Basic Magnesium Sulfate Cement (BMSC), penetration, experimental research, numerical simulation

## Abstract

The penetration resistance of the new material Basic Magnesium Sulfate Cement (BMSC) is studied through comprehensive application of an experimental and numerical simulation method. This paper consists of three parts. The first part introduces the preparation of Basic Magnesium Sulfate Cement Concrete (BMSCC) and the study of its dynamic mechanical properties. In the second part, on-site testing was carried out on both BMSCC and an ordinary Portland cement concrete (OPCC) target, and the anti-penetration performance of the two materials was analyzed and compared from three aspects: penetration depth, crater diameter and volume, and failure mode. In the last part, the numerical simulation analysis was carried out based on LS-DYNA, and the effects of factors, such as material strength and penetration velocity on the penetration depth, are analyzed. According to the results, the BMSCC targets have better penetration resistance performance than OPCC under the same conditions, mainly manifested in smaller penetration depth, smaller crater diameter and volume, as well as fewer cracks.

## 1. Introduction

Concrete is widely used as a structural material in modern civil engineering due to its high compressive strength, good durability, and strong ability to protect reinforcement. Currently, reinforced concrete structures are mostly adopted in protective engineering and fortifications among various countries. Therefore, researching the penetration effect of projectiles on concrete structures is essential. Over the past hundred years, scientists from various countries have conducted detailed research on the penetration for concrete materials by using the following three methods.

The first method is on-site testing. Due to the limitation of research conditions, initially the penetration resistance research of concrete materials was conducted in various countries, mainly by tests of field live projectile, and then a large amount of data were obtained and analyzed to derive corresponding empirical formulas, such as the Petry formula [1], the BRL formula [2], the ACE formula [3], the Berezan formula [4], and so on. With the improvement of laboratory technique, scholars from various countries have systematically studied the penetration resistance of concrete targets to different types of projectiles with materials, masses, shapes, and speeds through experiments. Among them, Frew [5] conducted penetration tests on concrete targets with pointed oval projectiles of different masses and materials, and they obtained non-dimensional penetration depth and velocity curves that coincide with different projectiles that have the same strength. Forrestal [6] had penetration tests on cement mortar and concrete targets with oval-shaped projectile head at different velocities. The variation rules of penetration depth with velocity of projectile were obtained. Meanwhile, in order to improve the penetration resistance of concrete, scholars from various countries carried out experimental research from three aspects: improving concrete strength, adding high-strength fiber, and using high-strength aggregates. Zhang [7] conducted penetration tests on concrete targets with different strengths by using the same projectile, and they found that the penetration depth and crater diameter of targets decrease with the increase in compressive strength of concrete. Sovják [8] and Máca [9] added steel fiber to the concrete, and they obtained the optimal amount of steel fibers that enhance the penetration resistance and the high-strength concrete that has the best ductility through experiments. Liu et al. [10] and Wu et al. [11] succeeded in making ultra high-performance concrete with ceramsite and corundum, respectively, instead of ordinary coarse aggregate, and they conducted detailed analyses of its dynamic response and penetration resistance. On-site testing is relatively simple, and the use of empirical formulas is convenient, which has played an important role in predicting the penetration ability of projectiles and the protection ability of targets. However, since the empirical formulas are based on specific projectiles, targets, and specific velocities, they are limited in applications. Besides, only the penetration status at the beginning and the end can be studied through tests, while the penetration process cannot be described in detail.

The second one is theoretical analysis. Theoretical analysis of concrete penetration caused by projectiles is mainly focused on building an engineering analysis model based on a single mechanism, of which the most representative is the cavity expansion approximation theory. Bishop [12] first proposed the expansion equations of quasi-static spherical and cylindrical cavities in a semi-infinite media, and the force on the projectile head was predicted when it penetrates metal targets by using the equations. Subsequently, Goodier [13] studied the issue of projectile penetrating metal plate by applying the spherical cavity expansion theory based on the cavity expansion theory. Butler and Rohani [14,15] researched the penetration of projectiles into rocks and concretes based on the cavity expansion theory and by taking advantage of the compressibility and shear properties of materials. Luk [16] first applied the dynamic spherical expansion theory to concrete penetration problems. Forrestal [17] proposed the Forrestal formula, based on this theory, combined with experimental data. He et al. [18] considered the shear–dilation effect of concrete in the dynamic spherical expansion theory, resulting in a better match between the calculated penetration depth and the measured results. Wu et al. [19] derived a cylindrical cavity expansion model based on an improved double shear strength criterion for analyzing the anti-penetration problem of granite targets. Zhang et al. [20] re-established the response partition of cavity expansion by considering the compression and expansion characteristics of concrete and established a dynamic spherical cavity expansion theory. The advantage of cavity expansion theory is that it can help analyze the simulation of complex projectile intrusion by using a simple and approximate concrete damage constitutive model. It is currently recognized as a relatively successful analytical method.

The third one is numerical simulation. In recent years, with the rapid development of computer technology and gradually matured calculation methods, numerical simulation has increasingly become the main mean of studying penetration problems. Currently, the HJC model and the RHT model are the commonly used concrete constitutive models. Holmquist et al. [21] proposed the HJC model, which can be applied to large strains, high strain rates, and high pressures, and they obtained 19 parameters in the HJC model of ordinary concrete, including basic mechanical parameters, strength and rate effect, equation of state, and damage, which are based on a large number of experimental data and numerical simulations. Kong et al. [22] improved the parameters and tensile damage model of the HJC model through extensive research and applied the improved model to numerical simulations of tunnel excavation and collapse, achieving good results. The RHT model was proposed by Riedel et al. [23], which can better describe the mechanical properties of concrete materials under different strain rates and loading conditions. Huang et al. [24] numerically simulated a projectile penetrating the concrete target and obtained good results, demonstrating that the RHT model is effective for simulating concrete failure modes dominated by crushing and destruction. Tu et al. [25] modified the residual strength surface, the ratio of tensile and compressive meridians, the strain rate effect of tensile strength, and the softening segment of the tensile stress–strain curve of the RHT model. Embedding these two constitutive models in LS-DYNA and AUTODYN, respectively, has been widely used by scholars all over the world.

MgO is commonly used as an expansive agent to improve the crack resistance of face slab concrete [26] and later became widely used in the production of magnesium-based matrix cementitious materials, such as magnesium oxychloride cement, magnesium oxysulfate cement, and magnesium phosphate cement, which have different hydration mechanisms, hydration products, and microstructures [27,28,29]. Compared with traditional calcium-based cementitious materials, reactive magnesia-based cementitious materials exhibit excellent mechanical properties. Basic Magnesium Sulfate Cement (BMSC) is a new type of magnesia cementitious material developed by Yu et al. [30]. It has the advantages of rapid solidification, early strength, high strength, high flexural strength, and high toughness because of a new phase 5Mg(OH)_2_·MgSO_4_·7H_2_O (also known as the 5·1·7 phase) [31]. Zhu et al. [32] compared and analyzed the static mechanical properties of BMSC concrete and ordinary Portland cement (OPC) concrete through experiments. It was found that, at the age of 90 days, the strength of magnesia sulfate cement concrete with the same strength was increased by 23~28% compared to ordinary concrete, and the splitting tensile strength was increased by 27~121%. Meanwhile, the raw material MgO for BMSC can be obtained from the waste of Mg(OH)_2_ produced in lithium extraction from salt lakes through a certain process of calcination. Another main raw material, MgSO_4_, is widely available in the byproducts of magnesium desulfurization method used in thermal power plants and steel plants. Therefore, compared with OPC, BMSC is more energy-saving and environmentally friendly. Currently, research on BMSC concrete mainly focuses on mix proportion design and static mechanical properties [33,34]. A systematic study of the anti-penetration performance of magnesia sulfate concrete is of great significance for its application in protective engineering.

In this study, on-site testings and numerical simulations were carried out on the projectile penetration of BMSC concrete and OPC concrete with different strength grades. The results, including penetration depth, crater diameter and volume, and failure mode, are analysed and discussed. These results show the influence of different cements, concrete strengths, and projectile velocities on the penetration performance of the target.

## 2. Test Preparation

### 2.1. Raw Materials

(1) BMSC: Main raw materials are calcined 50.6% Light-burned MgO (LBM), 25.4% industrial-grade magnesium sulfate heptahydrate (MgSO_4_·7H_2_O), 23.5% grade I fly ash (FA), and 0.5% core admixture (Citric acid, CA, USA). The physical and mechanical properties of the BMSC are listed in Table 1, and the chemical components of the LBM and FA are summarised in Table 2.

(2) Fine aggregate: river sand produced in Ganjiang was provided by Jiangsu Liyang Laijiang Concrete Co., Ltd.(Changzhou, China), with an apparent density of 2650 kg/m^3^, mud content of 1.4%, and fineness modulus of 2.6. It is classified as medium sand at the II district level.

(3) Coarse aggregate: crushed stone was provided by Anhui Xingyuan mineral Co., Ltd.(Anqing, China), with a particle content of 4.8% in faller gill, a crushability index of 10.4%, an apparent density of 2610 kg/m^3^, and a packing density of 1440 kg/m^3^. It is classified as 10~20 mm continuous gradation.

### 2.2. Specimens Preparation

Based on Table 3, three different strength grades of the BMSCC specimens were prepared and denoted as BMSC C30, BMSC C50, and BMSC C70. In order to show the difference between BMSCC and ordinary Portland cement concrete (OPCC), two different strength grades of the OPCC specimens were prepared and denoted as OPC C30 and OPC C50.

**Figure 1:**
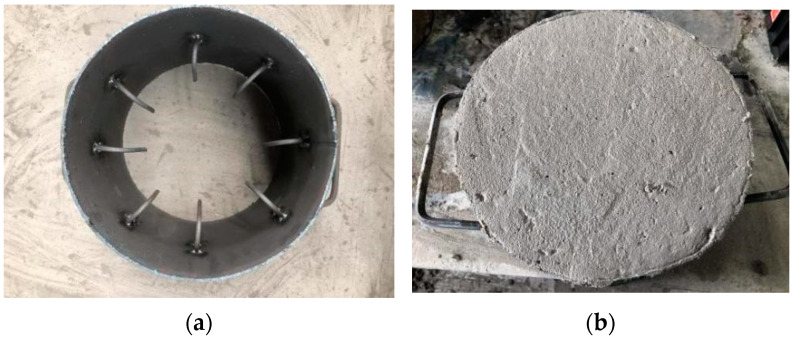
Targets of BMSC concrete. (**a**) Steel mold of the external side of target. (**b**) Specimen of target.

**Figure 2:**
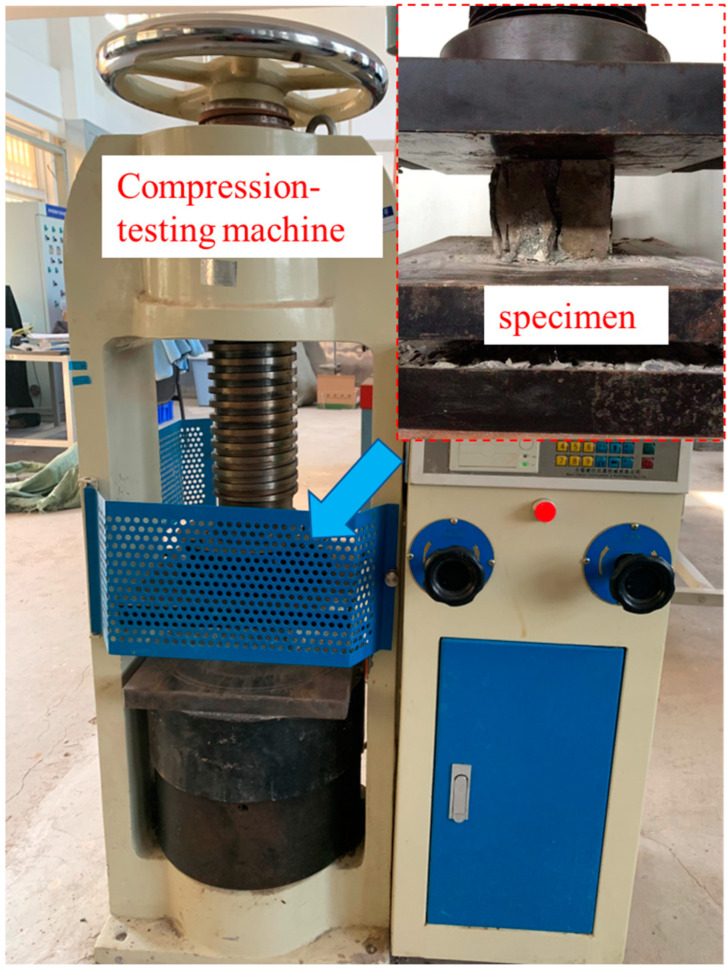
28d compressive strength test specimens and equipment.

Cylindrical targets measuring Ø30 × 25 cm were designed. The target adopts a 5 mm-thick steel hoop mold. The middle part in the height direction of the inner side of the steel hoop is welded to 4 pairs of L-shaped reinforcement symmetrically around the center of the circle. The long part is 10 cm, and the bending part is 4 cm (as shown in Figure 1a). The L-shaped reinforcement is used to strengthen the friction between the steel hoop and the target, aiming at preventing the target from sliding due to the impact force of the projectile. In addition, three cubic specimens with sizes of 100 mm × 100 mm × 100 mm are required for each mix proportion to measure the 28d compressive strength of concrete. (as shown in Figure 2).

All specimens would been compacted using a vibrating table after pouring was performed. The specimens were stored in an indoor environment at a temperature of 20 ± 2 °C and a relative humidity of 60 ± 5% for an additional 28 d for natural curing.

### 2.3. Test Device and Test Principle

**Figure 3:**
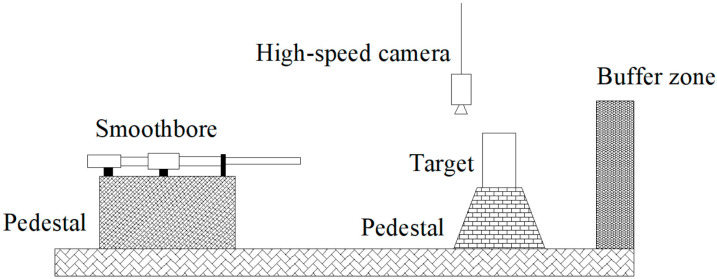
Schematic illustration for penetration test.

**Figure 4:**
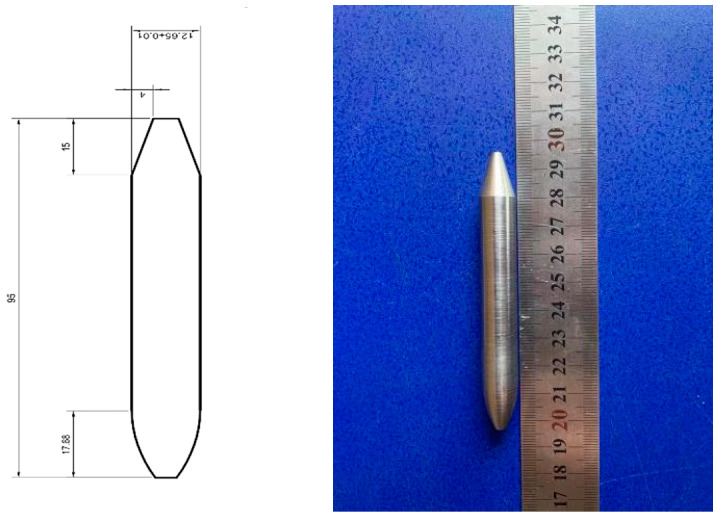
Projectile.

Figure 3 and Figure 4 depict the geometric model and size of the test device and penetration projectile used in this thesis. Specifically, the test device includes a 12.7 mm smooth-bore gun, target, a high-speed camera, and a buffer zone. The projectile is a high-strength tungsten alloy bullet, and its size is represented in Figure 3. The projectile has a diameter of 12.65 mm, a length of 95 mm, a density of 17.6 × 10^3^ kg/m^3^, a mass of 180 g, and a curvature radius–diameter ratio (CRH) of the head of 3.

To prevent the cylindrical target from rolling, a wooden wedge was used to secure it onto the target platform. This ensured that the distance between the smooth-bore gun’s barrel outlet and the back of the concrete targets remained fixed at 6 m. At the same time, the surface of the target is perpendicular to the barrel of the smooth-bore gun, and the axis of the barrel is aligned with the center of the target.

### 2.4. Test Results and Analysis

The initial velocity of the projectile is controlled by the charge. After the experiment, the residual velocity of the projectile is determined through high-speed photography. At the same time, the diameter, volume, and depth of the bullet hole on the surface of the target are measured, and the penetration depth is obtained through calculation. All experimental results are summarized in Table 4.

#### 2.4.1. Penetration Depth

**Figure 5:**
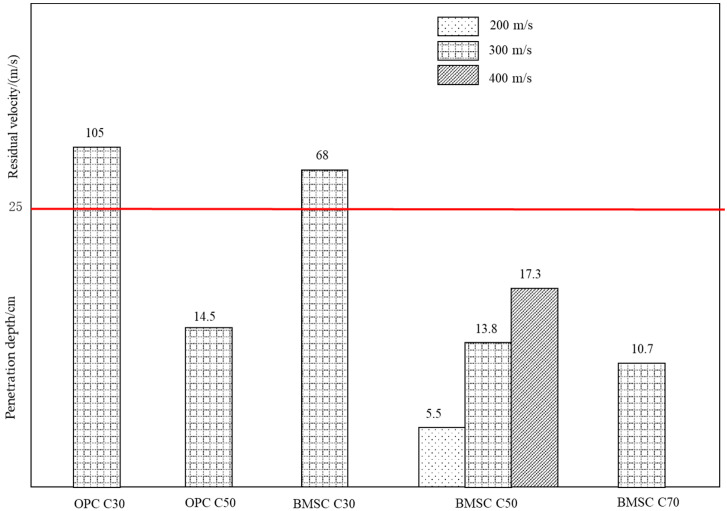
Comparison of penetration depths of concrete targets.

The penetration depth of target is an important basis for evaluating the penetration resistance of materials. The comparison of penetration depths of concrete targets with different strength grades has been shown in Figure 5. Based on Figure 5, it can be seen that, for BMSCC targets of the same strength, the greater the initial velocity of the projectile, the greater the penetration depth of the target. Besides, when the initial velocity of the projectile is the same, the penetration depth of the BMSCC target will decrease with increasing concrete strength. Therefore, it can be concluded that projectile velocity and concrete strength both have significant effects on the penetration resistance of the target. Meanwhile, when the initial velocity of the projectile is 300 m/s, it can be observed that the residual velocity of the projectile penetrating the BMSC C30 target with the same strength is smaller than that penetrating the OPC C30 target, and the penetration depth of the BMSC C50 target is also smaller than that of the OPC C50 target. This indicates that material properties also have an impact on the penetration resistance of concrete, and compared with the OPCC targets, the BMSCC targets have better penetration resistance performance under the same conditions.

#### 2.4.2. Crater Diameter and Volume

**Figure 6:**
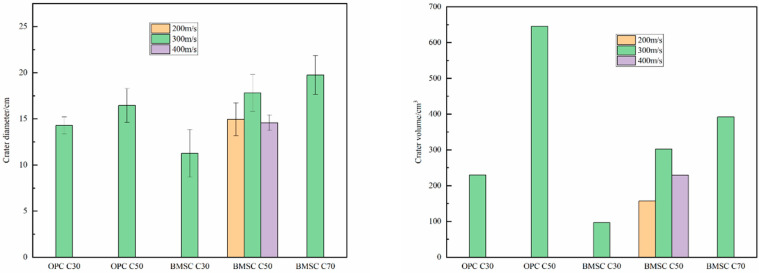
Comparison of crater diameter and volume of concrete targets.

Figure 6 shows the comparison of crater diameter and crater volume of concrete targets. The diameter of the crater is obtained by measuring the diameter in four different directions and taking the average. The volume of the crater is calculated by filling it with standard sand. Based on the Figure 6, for both the OPCC target and the BMSCC target, the crater diameter and volume increase with the increase in concrete strength when the projectile velocity is the same. This result is consistent with the experimental results obtained by Zhang [37] because, when the strength of the target increases, the interface pressure between the projectile body and the target will increase under the same velocity impact, resulting in greater surface damage. Meanwhile, under the same conditions, the crater volume of OPC C30 and OPC C50 targets is 2.38 and 2.14 times that of BMSC C30 and BMSC C50 targets, respectively. It can be concluded that BMSCC has a better ability to resist the surface damage of the target than OPCC.

#### 2.4.3. Analysis of Failure Mode of Target

**Figure 7:**
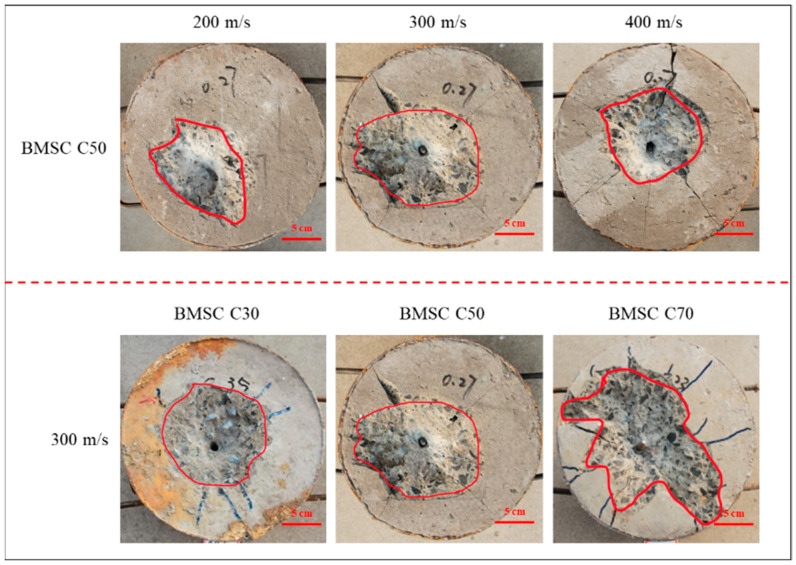
Failure modes of BMSCC targets with different strength and projectile velocity.

Figure 7 shows the failure mode of the BMSC C50 target surface under different projectile velocities. The craters are all funnel-shaped, with radial cracks at the edges, and the larger the velocity of the projectile, the more cracks and the larger the width. When the projectile velocity is 400 m/s, the cracks of the BMSC C50 target extend to the edge, causing some of the projectile’s energy to be absorbed by the steel barrel, resulting in a smaller diameter and volume of the crater than at 300 m/s (as shown in Figure 5). Meanwhile, Figure 6 also shows the failure mode of different strength BMSCC targets at the same velocity (300 m/s). The crater of the BMSC C30 target is more regular, and there is no obvious damage at the edge of the target. The surface area of damage in the BMSC C70 target is large, with multiple cracks, but the depth of projectile penetration is small, and some of the projectile is exposed outside.

**Figure 8:**
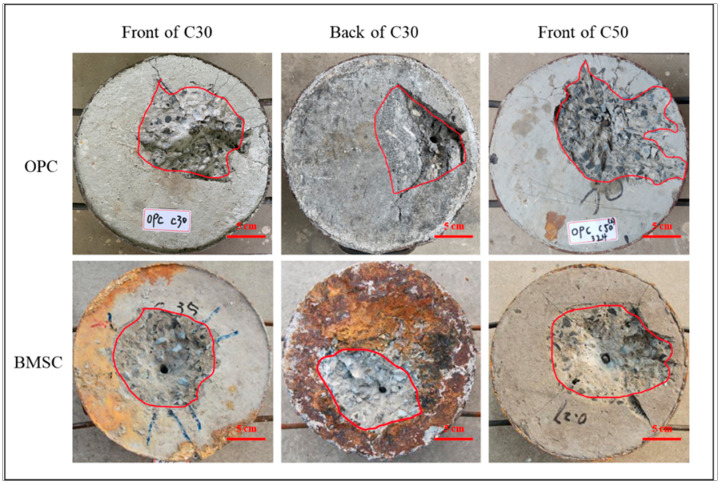
Failure modes of BMSCC and OPCC targets (projectile velocity = 300 m/s).

Figure 8 shows the failure mode of the BMSCC and OPCC target under the same projectile velocities. As shown in the figure, both the BMSC C30 and OPC C30 targets were penetrated by the projectile and damaged on the backside. The failure mode is similar to that on the front side, but the area is relatively smaller. Compared with the BMSC C30 target, the OPC C30 target not only has a larger damage area on the impact surface, but also has multiple cracks formed.

## 3. Numerical Simulation

### 3.1. Finite Element Modeling

**Figure 9:**
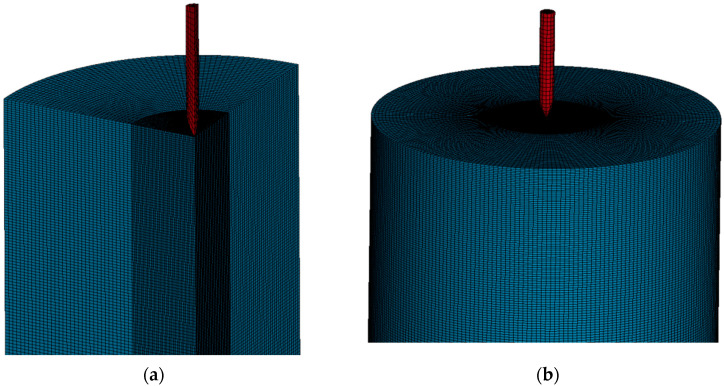
Numerical simulation model. (**a**) 1/4 calculation model. (**b**) Symmetrical full target model.

The LS-DYNA finite element pre-processing program was used for modeling, the basic unit of which is g-cm-μs. A Solid164 three-dimensional entity unit was used for projectile and concrete models. During the grid division process, grid cells were defined by their densities. As shown in Figure 9, fine grids were employed within a 5 cm-radius of the target center, while coarse grids were used on the periphery of the target. This approach ensured both the accuracy of the calculation results and a reduction in the number of cells, thereby properly speeding up the calculation.

### 3.2. Material Model and Parameters

The Johnson-Cook elastic–plastic model was selected for the projectile material, which can accurately describe the behavior of metal materials under large deformations, high strain rates, and high temperatures. For the concrete target, the isotropic Johnson-Holmquist-Concrete material model was adopted, which takes into account failure and is related to strain rate. Model parameters are shown in Table 5, Table 6 and Table 7.

### 3.3. Contact and Boundary Conditions

The contact–collision interface between the projectile and the target’s interface model is defined using the symmetric penalty method. The specific contact type is defined by using the keyword *CONTACT_ERODING_SURFACE_TO_SURFACE, where the projectile is the main surface, and the target is the secondary surface. As the concrete element is deleted after reaching the ultimate stress, a new contact surface will be defined inside the material. Based on practical test results, non-reflecting boundary conditions are set for the external and bottom surfaces of the cylindrical target, and symmetrical constraints are imposed on the 1/4 section of both the target and projectile.

### 3.4. Simulation Results and Analysis

#### 3.4.1. Process of Penetration

**Figure 10:**
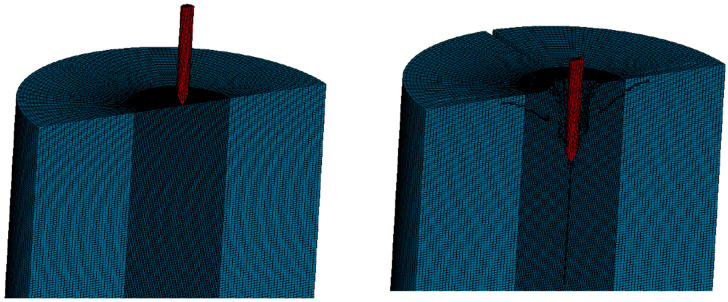

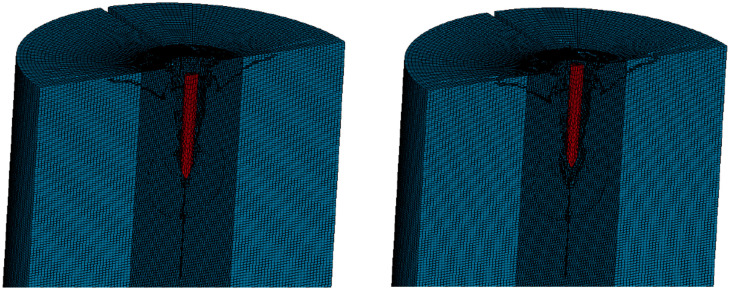
Penetration for BMSC C70 target. (projectile velocity = 300 m/s).

Figure 10 shows the numerical simulation of the penetration failure of BMSC C70 target with a projectile velocity of 300 m/s and a target height of 25 cm. During the penetration process, it can be observed that, after the bullet penetrates the target surface, the high-pressure area centered around the projectile head spalls and cells are deleted, and then the front pit area is formed. As the projectile penetrates deeper into the target, a tunnel pit is formed, with the projectile head being the most stressed area. When the projectile velocity reaches zero, the projectile has reached its maximum depth of penetration, and the pressure on the projectile head decreases. At this point, the whole projectile is squeezed by the concrete, the thrust of which will push the projectile backward along the tunnel pit.

#### 3.4.2. Variation Rules of Projectile Velocity

**Figure 11:**
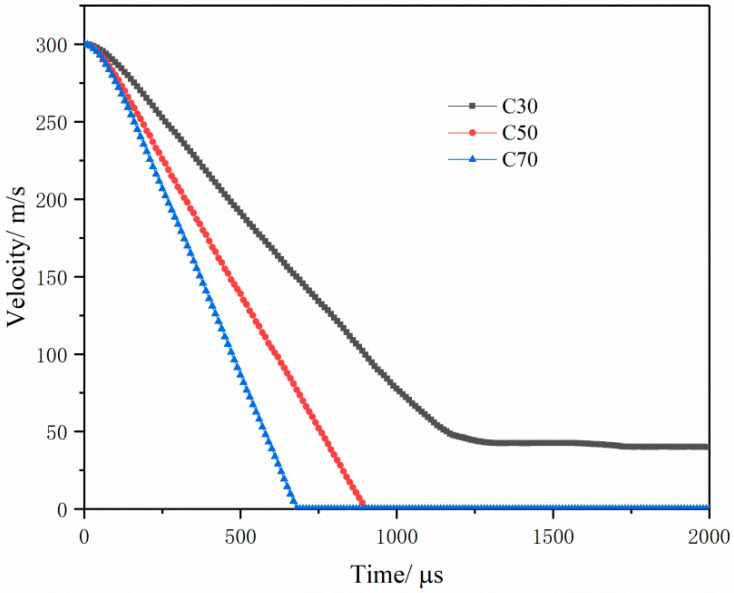
Time–history curve of projectile axial velocity during the penetration for BMSCC target.

Figure 11 denotes the time–history curve of projectile velocity during the penetration of the BMSCC target obtained through numerical simulation. The simulation results show that the bullet penetrates the target at a speed of 300 m/s. Due to the shallow contact with the target in the earliest stage, there is relatively little resistance, resulting in a relatively flat time–history curve of projectile velocity under the three working conditions at the beginning. However, as the projectile penetrates deeper, the projectile velocity begins to show linear attenuation with different slopes, and the more strength the BMSCC has, the faster the velocity attenuation will be. This suggests that the higher the strength of BMSCC, the greater the resistance against projectile penetration.

#### 3.4.3. Depth of Penetration

Numerical simulations were conducted for all test conditions of the BMSCC and OPCC target, and the comparison between the simulated values and experimental values is shown in Table 8. Based on the table, it can be seen that the error between the simulated and experimental values for OPCC target is −1.4% to 6.1%, and for the BMSCC target, it is 7.3% to 21.8%. This indicates that the model parameters used in the simulation can effectively simulate the penetration process of the projectile into the OPCC and BMSCC targets.

### 3.5. Influence Rules of Parameters

In order to study the variation of penetration depth of BMSC concrete with concrete strength and penetration velocity of projectile, the numerical simulation method was adopted in this paper. The target model is a cylinder with diameter of 30 cm and height of 50 cm. The parameters of the model and materials are shown in Table 5, Table 6 and Table 7.

#### 3.5.1. Impact of Concrete Strength

**Figure 12:**
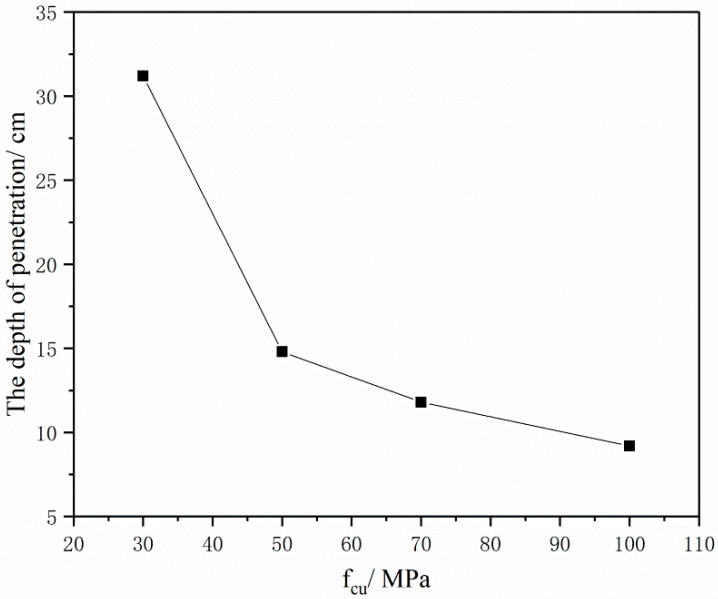
Relationship between the strength of the BMSCC target and the penetration depth (projectile velocity = 300 m/s).

Figure 12 shows the relationship between the strength of BMSCC concrete target and the penetration depth when the velocity of the projectile is 300 m/s. The figure indicates that the penetration depth of the target decreases with an increase in concrete strength. When the strength of BMSCC increases from 30 MPa to 70 MPa, the penetration depth reduces from 31.2 cm to 11.8 cm. However, when the strength increases from 70 MPa to 100 MPa, the penetration depth only reduces by 2.6 cm, which indicates that changes of target penetration depth become smaller with the increase in concrete strength. This further explains that, when the strength of concrete keeps increasing, its impact on the penetration resistance of concrete becomes smaller and smaller.

#### 3.5.2. Impact of Penetration Velocity

**Figure 13:**
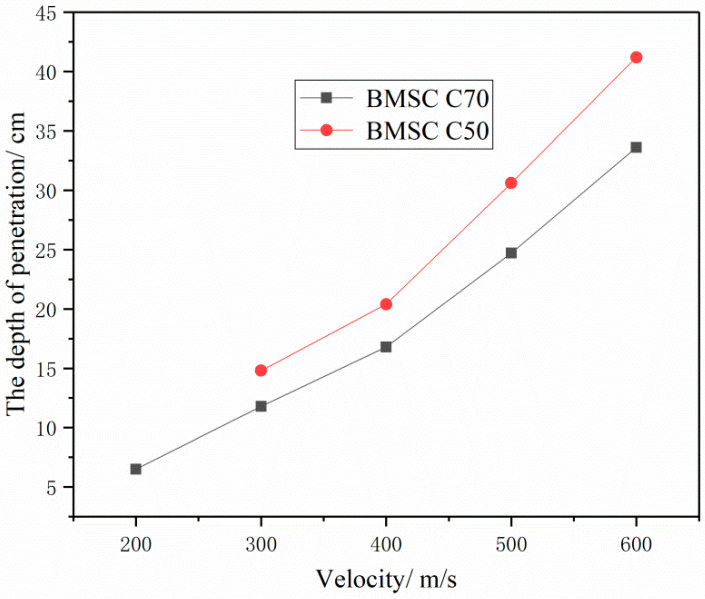
Relationship between the strength of the BMSCC target and the projectile velocity.

Figure 13 shows the relationship between the strength of the BMSCC target and the projectile velocity. It indicates that the penetration depth of the concrete target increases with an increase in projectile velocity, but there is no nonlinear relationship between them. Moreover, by comparing the penetration depths of the concrete targets with two strengths at different velocities, it can be concluded that the impact of projectile penetration velocity on the penetration depth of the target will be greater if the concrete strength is lower.

### 3.6. Comparison of Penetration Capability between OPCC and BMSCC

**Figure 14:**
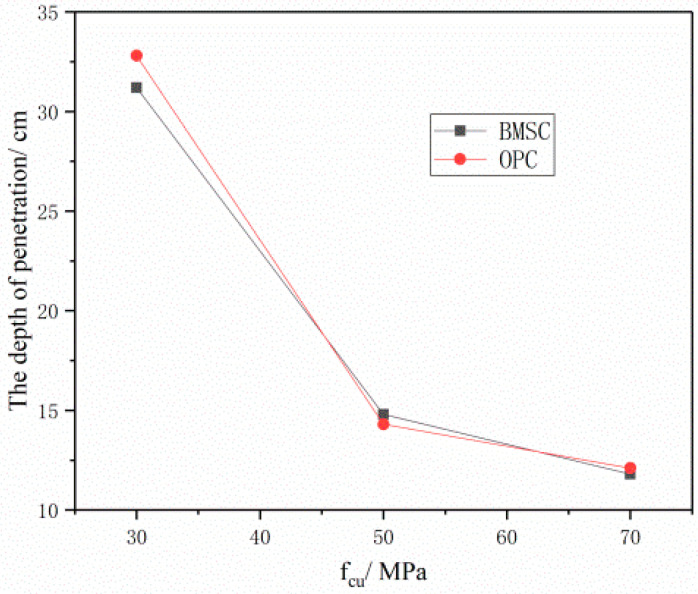
Comparison of penetration depth of concrete target at same projectile velocity (300 m/s).

**Figure 15:**
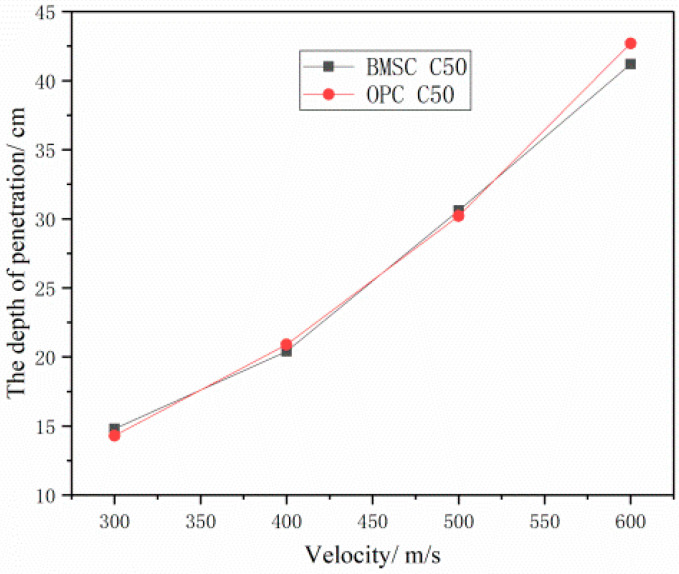
Comparison of penetration depth of C50 concrete target at different projectile velocities.

Figure 14 and Figure 15 illustrate the relationship between the penetration depth of two different target materials and the concrete strength and projectile velocity. The figures indicate that, under the same penetration conditions, there is little difference in the penetration depth between the BMSCC and OPCC target materials. However, when the concrete strength is C30 or the projectile velocity is 600 m/s, the penetration depth of the BMSCC target material is significantly smaller than that of the OPCC target material. This suggests that the anti-penetration performance of BMSCC is significantly better than that of OPCC when the concrete strength is low or the projectile velocity is high.

## 4. Conclusions

(1) The on-site test results have shown that projectile velocity and concrete strength both have significant effects on the penetration resistance of the BMSCC target. The greater the initial velocity of the projectile, the greater the penetration depth of the target. Besides, when the initial velocity of the projectile is the same, the penetration depth of the BMSCC target will decrease with increasing concrete strength.
(2) The crater diameter and volume increase with the increase in BMSCC strength when the projectile velocity is the same. When analyzing the failure mode of target, the craters are all funnel-shaped, with radial cracks at the edges, and the larger the velocity of the projectile, the more cracks and the larger the width.
(3) The model parameters shown in Table 5, Table 6 and Table 7 can effectively simulate the penetration process of the projectile into the OPCC and BMSCC targets.
(4) Material properties also have an impact on the penetration resistance of concrete, which is obtained through on-site testing and numerical simulations. The BMSCC targets have better penetration resistance performance than OPCC under the same conditions, mainly manifested in smaller penetration depth, smaller crater diameter, and volume, as well as fewer cracks.

## Figures and Tables

**Table 1:** Physical and mechanical properties of BMSC ([33]).

Cement	Standard Consistency/%	Initial Setting Time/min	Final Setting Time/min	Stability	Compressive Strength/MPa			Flexural Strength/MPa		
					3d	7d	28d	3d	7d	28d
BMSC	19.2	117	278	qualified	44.2	52.0	56.5	7.5	11.9	13.7

*Note: Definition of normal consistency is available in ASTM C219-20a, 2020* [35] *and details regarding the test method are available in ASTM C187-16, 2016* [36].

**Table 2:** Chemical components of FA and LBM.

Constituent/%	FA	Light Burned MgO
SiO_2_	54.88	2.87
Al_2_O_3_	26.89	0.12
CaO	4.77	1.34
MgO	1.31	87.3
Fe_2_O_3_	1.16	0.39
SO_3_	6.49	
I.L	3.1	6.56

**Table 3:** Mixing ratio and compressive strength of BMSCC and OPCC.

Strength Grate		Material Composition/kg·m^−3^					W/C	28d Compressive Strength/MPa
	Cementitious Material	Cement	FA	Coarse Aggregate	Fine Aggregate	Water		
BMSC C30	BMSC52.5	530		1078.3	718.6	185.5	0.35	36.2
BMSC C50	BMSC52.5	530		1078.3	718.6	143.1	0.27	51.5
BMSC C70	BMSC52.5	530		1078.3	718.6	121.9	0.23	67.4
OPC C30	PO52.5	430		1045	697	276	0.53	37.5
OPC C50	PO52.5	226	226	1132	679	158	0.35	55.1

**Table 4:** Test results of the penetration for concrete targets.

Concrete	Projectiles				Targets		
	Mass (g)	Charge (g)	Initial Velocity (m·s^−1^)	Residual Velocity (m·s^−1^)	Penetration Depth/cm	Crater Diameter/cm	Crater Volume/cm^3^
OPC C30	180.1	8.3	318	105	Penetrated	12.3	230
OPC C50	179.6	8.3	324	0	14.5	16.4	645.6
BMSC C30	179.7	8.3	297.1	68	Penetrated	13.95	96.7
BMSC C50	179.3	5.9	212.7	0	5.5	14.95	157.1
	179.7	8.3	297.2	0	13.8	17.80	302
	179.6	11.6	390	0	17.3	14.58	229.7
BMSC C70	180.2	8.3	286	0	10.7	19.75	392

**Table 5:** Parameters of projectile material model (Unit: g-cm-μs).

Symbols	Meaning	Value	Symbols	Meaning	Value
ρ	Density	17.6	D_1_	First fracture parameter	0.16
G	Shear modulus	1.36	D_2_	Second fracture parameter	3.13
A	Yield stress constant	1.5 × 10^−2^	D_3_	Third fracture parameter	−2.04
B	Strain hardening constant	1.7 × 10^−3^	D_4_	Fourth fracture parameter	0.007
N	Strain hardening index	0.12	D_5_	Fifth fracture parameter	0.37
C	Strain-rate dependence constant	1.6 × 10^−2^	C	Intercept distance of $u_{s}-u_{p}$ curve	4.569
M	Temperature-dependent index	1.0	S_1_	First gradient coefficient	1.49
T_m_	Melting temperature	1723	S_2_	First gradient coefficient	0.0
T_r_	Room temperature	294	S_3_	First gradient coefficient	0.0
C_v_	Specific heat	4.77 × 10^−6^	$\gamma_{0}$	Grüneisen coefficient	2.17
${\overset{\cdot }{\varepsilon }}_{0}$	Refer to strain rate	1.0 × 10^−6^	α	First corrections of $\gamma_{0}$	0.46

**Table 6:** Parameters of BMSC C70 (Unit: g-cm-μs).

RO	G	A	B	C	N	FC
2.45	0.213	0.79	1.6	0.007	0.61	7.0 × 10^−4^
T	EPS0	EFMIN	SFMAX	PC	UC	PL
5.5 × 10^−5^	1.0 × 10^−6^	0.01	7.0	2.3 × 10^−4^	5.62 × 10^−4^	0.0085
UL	D1	D2	K1	K2	K3	FS
0.1	0.04	1.0	0.85	−1.71	2.08	0

**Table 7:** Parameters of OPC C50 (Unit: g-cm-μs).

RO	G	A	B	C	N	FC
2.40	0.157	0.79	1.6	0.007	0.61	5.0 × 10^−4^
T	EPS0	EFMIN	SFMAX	PC	UC	PL
4.73 × 10^−5^	1.0 × 10^−6^	0.01	7.0	1.6 × 10^−4^	5.62 × 10^−4^	0.0085
UL	D1	D2	K1	K2	K3	FS
0.1	0.04	1.0	1.2	1.35	6.98	0

**Table 8:** Comparison of on-site test results and numerical simulation results for concrete target.

Concrete	Test Results	Numerical Simulation Results	Error
OPC C30 (300)	198 m/s	210 m/s	+6.1%
OPC C50 (300)	14.5 cm	14.3 cm	−1.4%
BMSC C30 (300)	232 m/s	252 m/s	+8.6%
BMSC C50 (200)	5.5 cm	6.7 cm	+21.8%
BMSC C50 (300)	13.8 cm	14.8 cm	+7.3%
BMSC C50 (400)	17.3 cm	19.8 cm	+14.4%
BMSC C70 (300)	10.7 cm	11.8 cm	+10.3%

*Note: When the target is not penetrated by the projectile, the result in the table is penetration depth, and when it is penetrated, the result is the change in velocity of the projectile.*

## Data Availability

The data presented in this study are available in Table 4, Table 5, Table 6, Table 7 and Table 8 and Figure 12, Figure 13, Figure 14 and Figure 15.
